# Comparison of short-term efficacy of two bipolar radiofrequency ablation forceps for rheumatic heart disease concomitant with atrial fibrillation

**DOI:** 10.3389/fcvm.2024.1335407

**Published:** 2024-04-22

**Authors:** Ning Zhang, Ming Hou, Bo Mei, Yong Liu, Ying-long Lai

**Affiliations:** ^1^ Department of Cardiothoracic Surgery, Dazhou Dachuan District People's Hospital (Dazhou Third People's Hospital), Dazhou, Sichuan, China; ^2^ Department of Cardiac Macrovascular Surgery, Affiliated Hospital of North Sichuan Medical College, Nanchong, Sichuan, China; ^3^ Department of Cardiovascular Surgery, Dazhou Central Hospital, Dazhou, Sichuan, China

**Keywords:** bipolar radiofrequency ablation forceps, rheumatic heart disease, atrial fibrillation, *Cox-Maze* procedure, short-term efficacy

## Abstract

**Background:**

Currently, the bipolar radiofrequency ablation forceps manufactured by *AtriCure* are the main instrument for surgical ablation in patients with rheumatic heart disease (RHD) concomitant with atrial fibrillation (AF). The bipolar radiofrequency ablation forceps by *Med-Zenith* has a greater advantage in price compared with *AtriCure*. However, few studies have been reported on the comparison of their clinical efficacy. The aim of this study is to compare the short-term clinical efficacy of the two ablation forceps for RHD concomitant with AF.

**Methods:**

Clinical data of 167 patients with RHD concomitant with AF admitted to the Department of Cardiac Major Vascular Surgery, Affiliated Hospital of North Sichuan Medical College, were retrospectively analyzed, and the restoration efficacy of sinus rhythm (SR) and cardiac function after surgery were compared with two ablation forceps.

**Results:**

The end-systolic diameter of the right atrium and the end-systolic diameter of the left atrium in the patients of both groups at each postoperative time point decreased compared with that of the preoperative period (*P* < 0.05), and the left ventricular ejection fraction started to improve significantly at 6 months after surgery compared with that of the preoperative period (*P *< 0.05). There was no difference between the two groups of patients in the comparison of the aforementioned indicators at different points in time (*P* > 0.05). At 12 months postoperatively, the SR maintenance rate in using the ablation forceps by *Med-Zenith* (73.3%) was lower than that for *AtriCure* (86.4%) and the cumulative recurrence rate of AF in using the *Med-Zenith* ablation forceps was greater than that for *AtriCure*.

**Conclusions:**

The two bipolar radiofrequency ablation forceps compared in the study are safe and effective in treating patients of RHD concomitant with AF, and the ablation forceps by *AtriCure* may be more effective in restoring SR in the short term.

## 1 Introduction

Atrial fibrillation (AF) has been the subject of focused research on cardiac arrhythmias for a long time, and is one of the most common arrhythmias in clinical practice today; especially when combined with rheumatic heart disease (RHD), the risk of stroke is tripled, causing a high rate of disability and death (1–3). The *Cox-Maze III* procedure was once considered the gold standard for surgical treatment of AF, with the recurrence rate of AF of less than 10% at long-term postoperative follow-up (4). After decades of development, the radiofrequency-based *Cox-Maze IV* procedure has gradually become the mainstream of AF surgical ablation (5). As the *Cox-Maze IV* concomitant with a rheumatic valve procedure has matured, the ablation devices used in the procedure are no longer limited to the *AtriCure* bipolar radiofrequency ablation forceps as the *Med-Zenith* bipolar radiofrequency ablation forceps have also gradually begun to be used in the clinic (6). Compared with the radiofrequency ablation forceps by *AtriCure*, those by *Med-Zenith* undoubtedly have more advantages in terms of price, but there are few reports in the literature on their comparative clinical efficacy. This study focuses on the short-term efficacy of the two bipolar radiofrequency ablation forceps for RHD concomitant with AF, and further evaluates the clinical value and application prospect of the ablation forceps by *Med-Zenith*, with the aim of providing different choices of intraoperative ablation devices and reducing the economic burden of patients.

## 2 Materials and methods

From September 2018 to December 2021, about 212 patients with RHD concomitant with AF underwent the rheumatic valve concomitant with *Cox-Maze IV* procedure in the Department of Cardiovascular Surgery, Affiliated Hospital of North Sichuan Medical College. The final selection of 167 patients was made strictly in accordance with the following inclusion exclusion criteria for this study.

### 2.1 Research grouping

The selected patients were divided into two groups, with 81 cases in the control group using the ablation forceps Isolator Synergy OLL2, by *AtriCure*, USA, and 86 cases in the observation group using the ablation forceps *MZ-RFK* by *Med-Zenith*, China.

### 2.2 Inclusion and exclusion criteria

#### 2.2.1 Inclusion criteria

(1) Meeting the diagnostic criteria of RHD concomitant with AF in the 2017 *HRS/EHRA/ECAS* Guidelines (7); (2) age ≥ 18 years; (3) intraoperative use of either of the two bipolar radiofrequency ablation forceps (*Med-Zenith* or *AtriCure*).

#### 2.2.2 Exclusion criteria

(1) Combination of other cardiac diseases, such as infective endocarditis; (2) end-systolic diameter of the left atrium (LAESD) ≥ 70 mm; (3) previous ablation of AF or rheumatic valve procedure; (4) combination of hepatic and renal failure (requiring dialysis treatment); (5) stroke within the last 6 months and acute myocardial infarction within the last 6 weeks; (6) combination of other cardiovascular surgeries during the same period, such as coronary artery bypass grafting, ascending aortic replacement, or plasty; and (7) irregular intake of medication after the operation and significant review data missing (including in-hospital death).

### 2.3 Surgical techniques

**Figure 1:**
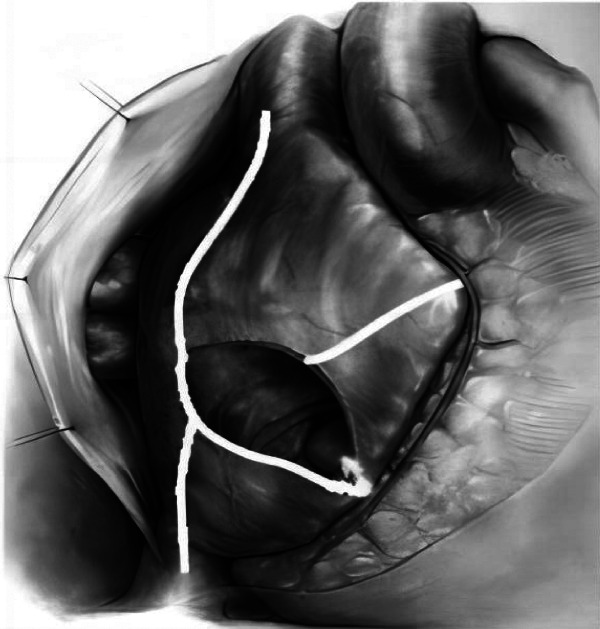
Modified maze surgical ablation route of the right atrium.

**Figure 2:**
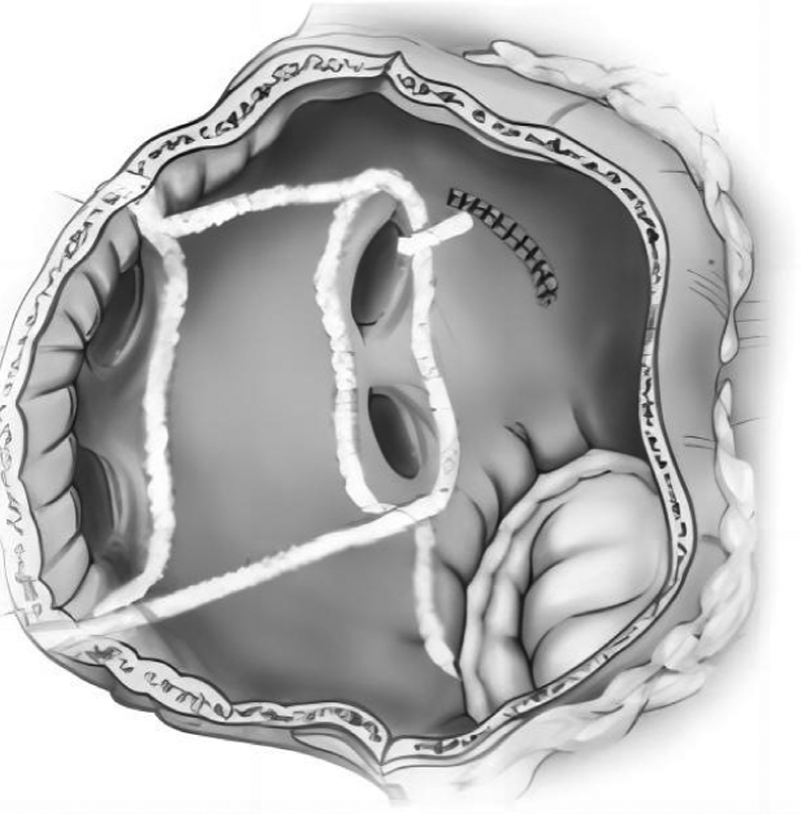
Modified maze surgical ablation route of the left atrium.

All the selected patients were operated by the same group of doctors. After general anesthesia, the chest of each patient was opened by a median sternal incision, heparinization was routinely performed at 3 mg/kg, the pericardium was incised in an inverted T-shape, the extracorporeal circulation was routinely established by the ascending aorta—upper and lower vena cava veins, and after the circulation was cooled down, the aorta was blocked, and radiofrequency ablation was performed at the root of the right auricle: the right atrium was incised obliquely, and radiofrequency ablation was performed successively at the right atrial incision to the superior vena cava line, the right atrial incision to the inferior vena cava line, the right atrial incision to the right auricle, the right atrial incision to the tricuspid annulus, and the right atrial incision to the coronary sinus line (Figure 1). The atrial septum was incised, and radiofrequency ablation was performed in the lines from the right superior pulmonary vein to the left superior pulmonary vein, the right inferior pulmonary vein to the left inferior pulmonary vein, the right inferior pulmonary vein to the medial posterior mitral annulus, the left inferior pulmonary vein to the left auricle, the left superior pulmonary vein to the left auricle, and the left superior and inferior pulmonary veins (Figure 2). Finally, ablation was performed at the root of the left auricle, the left auricle was ligated or excised, the ligament of Marshall was cut off, and the valve surgery was performed after the completion of ablation; the patient was routinely placed with temporary epicardial pacing wires during the operation, and a temporary pacemaker was connected after the operation (ablation of each pathway was performed four times until the wall was transmuted, and the radiofrequency ablation pen was added to deal with the isthmus lesion).

### 2.4 Postoperative management

(1) Postoperatively, amiodarone 450 mg + 5% dextrose 45 ml was routinely pumped intravenously (2 ml/h, the speed was adjusted according to the heart rate), and it was changed to oral amiodarone after extubation of the tracheal tube, the first 200 mg t.i.d. × 7 days, the dosage was reduced to 200 mg b.i.d. × 7 days, and the last oral amiodarone (200 mg q.d.) was discontinued until 3 months after the operation (the drug was discontinued when the heart rate was <60 beats/min and the QTc was > 500 ms); postoperatively, long-term anticoagulant of warfarin was given, and no other antiarrhythmic drugs (AADs) were generally added during the treatment period, and the long-term oral angiotensin-converting enzyme inhibitors (ACEIs) were administered after discharge from the hospital.
(2) All patients will have regular postoperative follow-ups in our outpatient clinic for at least 1 year: Rechecking 12-lead or 24-h ambulatory electrocardiogram (ECG) at the time of discharge from the hospital, and at 3/6/9/12 months postoperatively; and cardiac ultrasound at the time of discharge from the hospital, and at 6/12 months postoperatively (note: discharged patients should be informed that if they are unable to come to the hospital for follow-up examination or experience palpitations after the operation, they can undergo the relevant examinations nearby and inform their physicians of the results at the next follow-up examination).

### 2.5 Recurrence of AF

Any atrial arrhythmia greater than 30 s, including atrial fibrillation, atrial flutter, and atrial tachycardia, detected by 12-lead or 24-h ambulatory ECG, without the use of AADs (AF occurring within 3 months after surgery was not included as a recurrent AF event in this study).

### 2.6 Statistical analysis

All statistical analyses were performed with *SPSS* software (*version 26.0*; *SPSS Inc*., *Chicago, IL, USA*). The missing values were handled by the *K-Nearest Neighbors* algorithm. Categorical variables are expressed as frequencies and percentages, and continuous variables are expressed as the mean ± standard deviation. Median and interquartile range was used for non-normally distributed data.

(1) Two independent samples *t* test was used for comparisons between the groups for continuous variables, and *Wilcoxon* test was used for non-normally distributed data.
(2) Comparisons between groups for categorical variables were performed using the *χ^2^* test (chi-square Pearson test for minimum expected counts T ≥ 5, chi-square continuity correction for 1 ≤ T < 5, and chi-square Fisher's exact method for T < 1).
(3) Repeated-measures continuous variables were analyzed by repeated-measures *ANOVA*, and two-by-two comparisons at different time points were performed using the *Least-Significant Difference* (*LSD*)-*t* test.
(4) The variables from univariate analyses (*P* < 0.05) were jointly included in multivariate binary *logistic* regression, and the results were expressed using corrected odds ratio (OR) [95% confidence interval (CI)].
(5) The *Kaplan–Meier* survival analysis describes the cumulative recurrence rate of AF at 1 year postoperatively, and the results were subjected to the *Log-Rank* test.

## 3 Results

### 3.1 Baseline characteristics

As provided in Table 1, a total of 110 mitral valve replacements, 8 aortic valve replacements, 43 aortic combined valve mitral valve replacements, and 6 mitral valve repairs were performed in the two groups of patients; 165 cases of tricuspid valvuloplasty were performed during the same period. The control group and the observation group differed only in terms of previous coronary artery disease, preoperative oral warfarin, number of valves, and lactate level (*P *< 0.05), and there was no statistical difference between the two groups in terms of cardiac function class, LAESD, ablation time, duration and type of AF, etc. (*P *> 0.05).

**Table 1:** Comparison of baseline data between the two groups [$\bar{x}$ ± *s*, *n* (%), *M*(*P*_25_, *P*_15_) $\bar{x}$ ± s, *n* (%), *M*(*P*_25_, *P*_15_)].

Variable	Control group (*n* = 81)	Observation group (*n* = 86)	*t/Z/χ^2^* value	*P-*value
Age (years)	56.85 ± 8.65	57.62 ± 8.20	−0.586^a^	0.558
Gender			0.011^b^	0.915
Male	27 (33.3)	28 (32.6)		
Female	54 (66.7)	58 (67.4)		
BMI (kg/m^2^)	22.31 ± 3.06	22.87 ± 3.46	−1.108^a^	0.27
Course of AF			−0.931^c^	0.352
Course < 1month	14 (17.3)	19 (22.1)		
1 month ≤ course < 6 months	22 (27.2)	27 (31.4)		
6 months ≤ course < 12 months	13 (16)	9 (10.5)		
Course > 12 months	32 (39.5)	31 (36)		
Types of AF			1.512^b^	0.47
Paroxysmal	12 (14.8)	18 (20.9)		
Persistent	45 (55.6)	48 (55.8)		
Long-range persistent	24 (29.6)	20 (23.3)		
Diabetes	9 (11.1)	9 (10.5)	0.018^b^	0.893
Hypertension	11 (13.6)	11 (12.8)	0.023^b^	0.88
Coronary heart disease	6 (7.4)	18 (20.9)	6.199^b^	0.013
NYHA class			−0.439^c^	0.661
I	0	0		
II	26 (32.1)	29 (33.7)		
III	49 (60.5)	53 (61.6)		
IV	6 (7.4)	4 (4.7)		
Preoperative oral medications				
Warfarin	21 (25.9)	40 (46.5)	7.624^b^	0.006
ACEIs	31 (38.3)	34 (39.5)	0.028^b^	0.867
AADs	68 (84)	75 (87.2)	0.36^b^	0.549
Hb (g/L)	133 (123.5–141)	129.51 ± 17.58	−1.47^c^	0.141
ALT (U/L)	25 (16–41)	23 (15.75–37.25)	−0.918^c^	0.359
Cr (μmol/L)	67.4 (58.1–79)	68.55 (63.43–82.7)	−1.295^c^	0.195
Lactic acid (mmol/L)	2.84 ± 0.89	2.5 (1.9–3.3)	−2.048^c^	0.041
ALB (g/L)	38.52 ± 4.14	39.6 (37.7–41.18)	−1.739^c^	0.082
RAESD (mm)	51.4 ± 7.71	51.8 ± 6.97	−0.359^a^	0.72
LAESD (mm)	51.64 ± 7.68	51.68 ± 7	−0.039^a^	0.969
LVEDD (mm)	50.36 ± 8.65	49.13 ± 7.42	0.988^a^	0.325
LVEF (%)	57.36 ± 8.27	57.28 ± 6.07	0.08^a^	0.936
Numbers of valves replaced			6.399^b^	0.011
Single valve	53 (65.4)	71 (82.6)		
Double valves	28 (34.6)	15 (17.4)		
CPB time (min)	161 (135–198)	153.5 (128.75–185.5)	−1.31^c^	0.19
Aortic block (min)	98 (87.5–150)	98 (84.75–122.25)	−1.283^c^	0.2
Ablation time (min)	25.13 ± 3.33	25.15 ± 3.01	−0.049^a^	0.961

BMI, body mass index; NYHA, New York Heart Association; Hb, hemoglobin; ALT, alanine transaminase; Cr, creatinine; ALB, albumin; ACEIs, angiotensin-converting enzyme inhibitors; AADs, antiarrhythmic drugs; RAESD, end-systolic diameter of right atrium; LAESD, end-systolic diameter of left atrium; LVEDD, left ventricular end-diastolic diameter; LVEF, left ventricular ejection fraction; CPB, cardiopulmonary bypass.

^a^ *t* test.

^b^ *Wilcoxon* test.

^c^ *χ^2^* test.

### 3.2 Confounding factor correction

To exclude the interference of related confounding factors, we included postoperative AF recurrence as the dependent variable, and included the aforementioned factors (*P* < 0.05) together with the grouping variable (different ablation forceps) in the binary *logistic* regression model for multifactorial analysis. As provided in Table 2, after correcting for the aforementioned influences, the grouping variable remained (*OR *= 2.345, 95% *CI*: 1.117–4.923, *P *= 0.024) an independent influence on postoperative AF recurrence.

**Table 2:** Multifactorial *Logistic Regression Analysis* of AF recurrence after the rheumatic valve procedure concomitant with *Cox-Maze IV* procedure.

Variable	*β* value	Wald value	Corrected OR (95%CI)	*P-*value
Lactic acid (mmol/L)	0.127	0.507	1.136 (0.8–1.611)	0.476
Preoperative oral warfarin	0.311	0.715	1.364 (0.644–2.802)	0.398
Coronary heart disease	−1.025	2.917	0.359 (0.111–1.163)	0.088
Single valve	−0.004	0.0001	0.996 (0.441–2.250)	0.993
The bipolar radiofrequency ablation forceps (*Med-Zenith*)	0.852	5.073	2.345 (1.117–4.923)	0.024

### 3.3 Postoperative cardiac function

**Figure 3:**
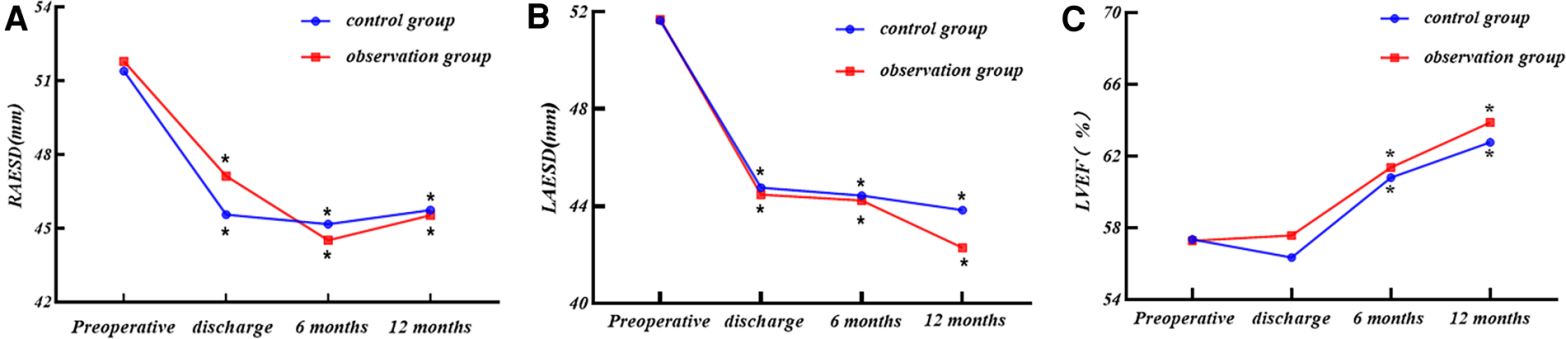
(**A**) RAESD, (**B**) LAESD, and (**C**) LVEF changes at each time point for the two groups. Comparison within group: compared with preoperative period. *P < 0.05.

We analyzed the end-systolic diameter of the right atrium (RAESD), LAESD, and left ventricular ejection fraction (LVEF) of the two groups at four time points: preoperatively, at discharge, at 6 months postoperatively, and at 12 months postoperatively by repeated-measures *ANOVA*, and performed simple effects analysis (two-by-two comparison), as presented in Table 3 and Figure 3.

**Table 3:** Comparison of cardiac ultrasound indices between the two groups ($\bar{x}$ ± *s*).

Variable	Groups	Preoperative	time of discharge	6 months postoperatively	12 months postoperatively
RAESD (mm)	The control group	51.40 ± 7.71^b,c,d^	45.56 ± 6.75^a^	45.17 ± 6.92^a^	45.75 ± 6.63^a^
	The observation group	51.80 ± 6.97^b,c,d^	47.13 ± 6.97^a,c,d^	44.52 ± 6.14^a,b^	45.53 ± 6.06^a,b^
	*P*-value	0.72	0.141	0.521	0.824
LAESD (mm)	The control group	51.64 ± 7.68^b,c,d^	44.75 ± 6.42^a^	44.44 ± 6.98^a^	43.84 ± 7.01^a^
	The observation group	51.69 ± 7^b,c,d^	44.48 ± 5.71^a,d^	44.24 ± 5.69^a,d^	42.29 ± 4.81^a,b,c^
	*P*-value	0.969	0.769	0.839	0.096
LVEF (%)	The control group	57.36 ± 8.27^c,d^	56.35 ± 7.96^c,d^	60.8 ± 7.23^a,b,d^	62.77 ± 8.06^a,b,c^
	The observation group	57.28 ± 6.07^c,d^	57.58 ± 7.65^c,d^	61.37 ± 7.47^a,b,d^	63.88 ± 6.81^a,b,c^
	*P*-value	0.936	0.308	0.618	0.333

Comparison within group: compared with preoperative time point.

^a^ (*P *< 0.05); compared with discharge.

^b^ (*P *< 0.05); compared with 6-month postoperative time point.

^c^ (*P *< 0.05); compared with 12-month postoperative time point.

^d^ (*P *< 0.05).

#### 3.3.1 End-systolic diameter of the right atrium

(1) There was no statistical difference in the RAESD of the two groups of patients at each time point (*P* > 0.05), nor was there any statistical difference in the trend of RAESD of the two groups at each time point (*P* > 0.05).
(2) The RAESD of the two groups showed a decreasing trend with the increase of time (*P *< 0.05).
(3) The RAESD decreased in both groups at all time points after surgery compared with the preoperative period (*P *< 0.05).

#### 3.3.2 End-systolic diameter of the left atrium

(1) There was no statistical difference in the LAESD of the two groups of patients at each time point (*P* > 0.05), nor was there any statistical difference in the trend of the LAESD of the two groups at each time point (*P* > 0.05).
(2) The LAESD of the two groups showed a decreasing trend with the increase of time (*P *< 0.05).
(3) The LAESD decreased in both groups at all time points after surgery compared with the preoperative period (*P *< 0.05).

#### 3.3.3 Left ventricular ejection fraction

(1) There was no statistical difference in LVEF of the two groups of patients at each time point (*P* > 0.05), nor was there any statistical difference in the trend of LVEF of the two groups at each time point (*P* > 0.05).
(2) The LVEF of the two groups showed the upward trend with the increase of time (*P *< 0.05).
(3) There was no significant difference in postoperative LVEF of the two groups at the time of discharge compared with the preoperative period (*P* > 0.05), and it started to improve significantly at 6 months postoperatively compared with the preoperative period (*P* < 0.05).

### 3.4 Postoperative rhythm

#### 3.4.1 Maintenance rate of sinus rhythm

**Figure 4:**
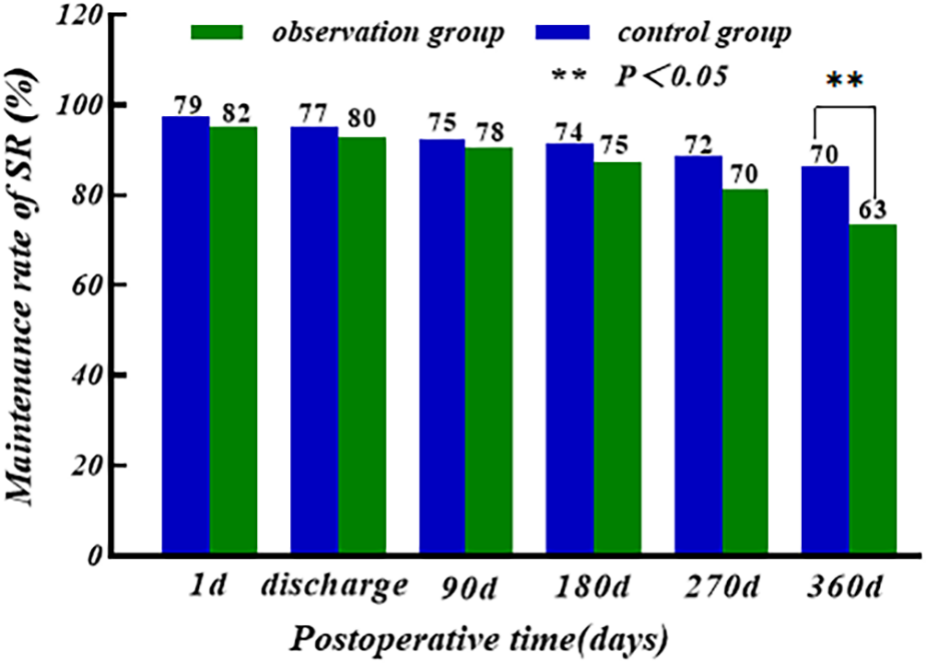
Comparison of SR between two groups after operation.

The sinus rhythm (SR) maintenance rate of the two groups showed a slow decreasing trend with the increase of time, and there was no significant difference in the first postoperative day, at the time of discharge, and at 3/6/9 months postoperatively (*P* > 0.05); the maintenance rate of SR of the control group (86.4%) was higher than that of the observation group (73.3%) at 12 months postoperatively, and the difference was statistically significant (*P* < 0.05), as shown in Figure 4.

#### 3.4.2 Cumulative recurrence rate of AF

**Figure 5:**
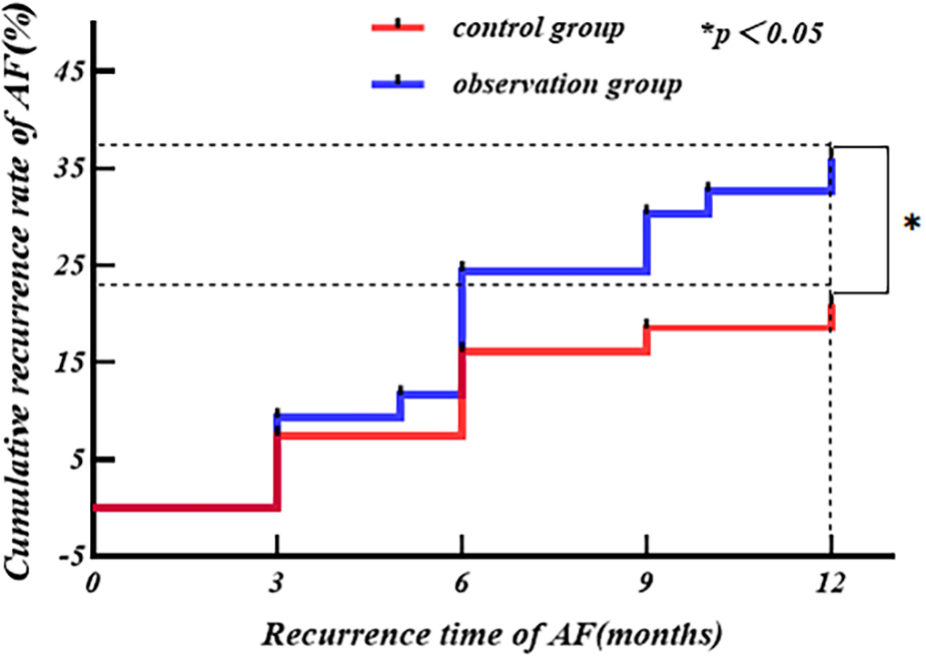
Cumulative recurrence rate of AF within 1 year after operation in the two groups.

The *Kaplan–Meier* survival analysis was performed with postoperative AF recurrence as the outcome end point event, with the independent variable being the grouping variable (different ablation forceps), and the time until the end point event was the time to AF recurrence (3 months ≤ time ≤ 12 months). The results revealed 31 cases of AF recurrence in the observation group and 17 cases of AF recurrence in the control group at 1 year after surgery. The cumulative recurrence rate of AF at 1 year after surgery in the observation group was higher than that in the control group (*Log-Rank*: *P *= 0.035), as shown in Figure 5.

## 4 Discussion

Atrial fibrillation, a common clinical arrhythmia, has been shown to be associated with the progression and worsening of heart failure, with the incidence of heart failure in patients with persistent and long-standing persistent AF being approximately 40%–55% (8, 9). In addition, the higher disability and mortality rates due to stroke in AF are a major global problem, and in particular, the risk of stroke is tripled in combination with RHD (1). Rhythm-control-based pharmacotherapy and catheter ablation were earlier proposed for the treatment of AF with the aim of preventing stroke, controlling heart rate, reducing symptoms, and improving the cardiac function and quality of life of patients (10–12). Unfortunately, drug therapy generally has serious side effects, and the stable maintenance of SR after catheter ablation often requires multiple ablations, both of which have not significantly improved patients’ left heart function and quality of life (13). Currently, the radiofrequency-based *Cox-Maze IV* procedure has replaced the original “cut-and-sew technique” as the main treatment for surgical ablation of AF, and the corresponding surgical ablation devices have also been developed rapidly (14). After decades of turnover, in the current domestic market the bipolar radiofrequency ablation forceps (*MZ-RFK*) manufactured by *Med-Zenith* are more frequently used in clinical application. Compared with the unipolar radiofrequency ablation device (unipolar linear ablation pen), the bipolar radiofrequency ablation forceps undoubtedly have a greater advantage. They have two parallel jaws, a curved upper jaw and a lower jaw, which can realize full and continuous contact between the electrode and the tissue when clamping the lesion tissue, creating a continuous ablation pathway and determining whether the tissue has reached complete wall permeability through the change of electrical conductivity. In addition, the parallel clamp design can avoid local energy concentration and reduce the damage to some low-impedance tissues (esophagus) (15).

Since the introduction of the first bipolar radiofrequency ablation forceps by *AtriCure* in 2000, after decades of development, the bipolar radiofrequency ablation forceps Isolator Synergy OLL2 have become the mainstay of surgical ablation applications for atrial fibrillation worldwide and are FDA approved surgical instruments for the surgical treatment of AF (16, 17). They provide radio frequency energy, and the two sets of electrodes ablate alternately, forming a columnar ablation line in the center without gaps, while parallel clamping closure, deformation, and pressure are consistent to ensure that ablation achieves galvanic isolation (18). The bipolar radiofrequency ablation clamp MZ-RFK independently developed by *Med-Zenith* Medical Devices, which was established in 2005 in China, together with its radiofrequency ablation generator MZ-RFG, can dynamically monitor 50 times/s of impedance and temperature changes when ablating the target tissue, and achieve precise wall penetration with the minimum effective power output (19). Relevant experimental data showed that bipolar radiofrequency ablation forceps heated the tissue with radiofrequency energy at 70–80°C for about 1 min to produce an ablation radius of 3–6 mm in depth, which is sufficient to achieve the required transmural effect for cardiac conduction block (20, 21). At present, it is not possible to say which of the two ablation forceps is superior in terms of wall penetration integrity, as there is no clear evidence that the created ablation trails completely block electrical conduction, and the surgeon's skill and left atrial size may affect wall penetration, which can only be assessed indirectly on the basis of the appropriate parameters. For further confirmation, perhaps a complete animal experimental design is needed to explore the tissue permeability of the two ablation forceps. It has been shown that the two ablation forceps with similar parallel clamping devices (embedded electrodes) can create similar ablation pathways and require roughly equivalent ablation times, which was confirmed by our results (22). The difference in the ablation time between the two groups was not significant, both taking an average of 25 min.

Although there is no direct evidence of a difference in the clinical efficacy of the two ablation clamps today, the present study found that the rate of SR maintenance at 12 months after the procedure was lower in the observation group (73.3%) than in the control group (86.4%). It has been reported in the literature that compared with the *Med-Zenith* ablation forceps, the two groups of bipolar electrodes of the *AtriCure* ablation forceps can transmit radiofrequency alternately at 264 cycles/s, forming a cross-electrode network to avoid deep tissue fissures leading to failure of ablation; the two groups of bipolar electrodes work in an alternating manner, leaving an intermittent period of electrocoagulation, so that there will not be a case of overheating of the ablated tissues that will cause the ablated tissues to become charred and deformed under continuous work, and the alternating emission can allow the impedance to rise slowly to reduce the loss of the radiofrequency energy transmitted to the deep tissues to make the tissues more likely to achieve a full permeability of the wall (23). We further found by the *Kaplan–Meier* survival analysis that the cumulative incidence of AF in the observation group was greater than that in the control group at 1 year post procedure (*P* < 0.05), suggesting that the *AtriCure* bipolar radiofrequency ablation forceps were indeed more effective in restoring and maintaining SR in the short term compared with the *Med-Zenith* ablation forceps. In addition, there was no significant difference in the rate of SR maintenance in the early postoperative period between the two groups in this study, which could not be separated from the role of oral amiodarone in the early postoperative period. Relevant studies have confirmed that amiodarone, as a Class III AADs, can significantly reduce the recurrence of AF in the early postoperative period (≤3 months) after surgical ablation of AF, but whether there is an effect on recurrence in the distant postoperative period has not yet been clearly confirmed (24, 25). The mechanisms by which amiodarone reduces early recurrence after ablation of AF include: (1) inhibition of autoregulation in the sinus node and atrioventricular junction area, slowing atrioventricular node and atrioventricular bypass conduction; (2) prolongation of the myocardial tissue action potential and the effective period of inactivity to eliminate atrioventricular refractoriness and to reverse the electrical remodeling of AF. We also found that the SR maintenance rate tended to decrease slowly with time in both groups, and a number of studies have also found that the rate of postoperative AF recurrence increases progressively with time. The cause of long-term recurrence may be the fading of scar trails isolating pulmonary veins leads to restoration of electrical conduction, ectopic origins of the triggering foci, and alterations of cardiomyocyte stroma (26, 27).

The results of repeated-measures *ANOVA* in this study showed that there were statistically significant differences in LAESD and RAESD of the two groups at each postoperative time point compared with the preoperative time point; LVEF significantly began to improve at 6 months postoperatively (*P* < 0.05); there were no statistically significant differences in intergroup comparisons between the two groups for LAESD, RAESD, and LVEF at each time point, and trends in the aforementioned indicators also did not differ between the two groups. This indicates that regardless of the kind of ablation forceps used, cardiac function improved in both groups of patients treated with the rheumatic valve procedure concomitant with *Cox-Maze IV* procedure, and it was a slow and long-term process. The relevant literature has shown that the rheumatic valve procedure can correct organic valve pathology, restore patients’ hemodynamics, reduce cardiac load, and significantly improve cardiac function (28, 29). It has been found that the *Cox-Maze* procedure can help restore the contractile function of the left atrium, reduce the load on the left atrium, avoid further dilatation of the left atrium, and improve the patient's cardiac function while reversing SR (30). This study also found that the postoperative LVEF of both groups did not improve much at the time of discharge from the hospital compared with the preoperative period, and it has been shown that the early postoperative LVEF after the rheumatic valve procedure concomitant with *Cox-Maze IV* procedure does not improve significantly compared with the preoperative period, and even decreases 10%, which may be a result of the combination of several factors: (1) preoperative valve disease leads to long-term overload of the left ventricle, myocardial persistent damage, and contractile function being severely impaired; (2) early postoperative cardiomyocyte edema, which affects myocardial fibers to regulate themselves abnormally, the myocardial contractile force is not fully restored to reach the optimal initial length, and left ventricular ejection volume may even be reduced (31). Pericardial tamponade and cardiac perforation rupture did not occur during the perioperative period in either group of patients in this study. Two cases of Degree III atrioventricular block were seen in the observation group and one case in the control group, both of which underwent permanent pacemaker implantation, which is not much different from the results of previous studies (32).

Nowadays, surgical ablation of AF is developing in the direction of being non-beating and minimally invasive. Mei's minimally invasive ablation technique created by Prof. Meiju in China utilizes TV thoracoscopy to ablate AF in a non-beating heart through a self-designed ablation line, which improves the success rate of the operation and reduces the occurrence of postoperative complications (33). Recently, given the close collaboration between cardiac surgeons and electrophysiologists, a one-stop sequential ablation strategy based on surgical/catheter ablation has been introduced with good results in patients with persistent AF. A related study demonstrated that this hybrid ablation model combines the advantages of intracardiac catheter ablation and epicardial surgical ablation, and to some extent can overcome the shortcomings of a single ablation technique (34). The rapid development of AF ablation technology has also led to innovations in ablation devices. Recently, the Three-dimensional Electronic Anatomical Marking System (*CARTO®^3^ Version7*) has been developed by Johnson & Johnson to quickly and accurately perform endocardial and epicardial marking to construct a three-dimensional model of the heart through a new marking catheter (*OCTARAY™*), as well as marking intracardiac electrical signal conduction, locating the foldback loop, and determining the target range of ablation (35). It is believed that in the future, a variety of advanced ablation devices will appear one by one, bringing the treatment of AF into a new era.

The present study was conducted to evaluate the clinical value and application prospect of the ablation forceps by *Med-Zenith*, and to provide some clinical basis for the selection of intraoperative ablation forceps for patients with RHD concomitant with AF in developing countries, but there are limitations in this study. This is a retrospective study, the baseline data of the two groups still have differences in a few factors; only 1-year postoperative examination data of the patients were collected, the number of included cases was small, and the postoperative ECG only recorded the results of a few time points, which can roughly assess the short-term efficacy of the two types of ablation forceps, while the medium-term and even long-term efficacy need to be further investigated.

## 5 Conclusions

In patients with RHD concomitant with AF who undergo the rheumatic valve concomitant with *Cox-Maze IV* procedure, the bipolar radiofrequency ablation forceps by *AtriCure* are indeed more effective in restoring SR in the short term. However, to a certain extent, the bipolar radiofrequency ablation forceps by *Med-Zenith* can be used to achieve similar results and reduce the financial burden on the patient.

## Data Availability

The original contributions presented in the study are included in the article/**Supplementary Material**, further inquiries can be directed to the corresponding author.
